# What Affects Quality of Life for People with Type 1 Diabetes?: A Cross-Sectional Observational Study

**DOI:** 10.3390/ijerph18147623

**Published:** 2021-07-17

**Authors:** Mi-Kyoung Cho, Mi-Young Kim

**Affiliations:** 1 Department of Nursing Science, Chungbuk National University, 1 Chungdae-ro, Seowon-gu, Cheongju 28644, Korea; ciamkcho@gmail.com; 2 College of Nursing, Hanyang University, 222 Wangsimni-ro, Seongdong-gu, Seoul 04763, Korea

**Keywords:** quality of life, self-efficacy, acceptance, type 1 diabetes mellitus

## Abstract

This study investigated the association between the quality of life (QOL) and type 1 diabetes mellitus (DM), a lifelong disease that requires constant management. A complex set of factors influence the QOL of people with type 1 DM, and understanding these factors requires further research. This research is a cross-sectional descriptive study. A survey on related variables such as acceptance of disease and efficacy for self-management of diabetes, was conducted among 111 participants with type 1 DM. The collected data were analyzed using PASW Statistics program, and factors influencing participants’ QOL were identified through hierarchical multiple regression. The study followed the Guidelines of Systematic Reporting of Examination in the STROBE checklist. The results showed that four variables exerted a significant effect on QOL (blood glucose level at hypoglycemia and complications in Model 1; efficacy for self-management of diabetes and acceptance and action in Model 2), and all the variables explained a majority of the variance in QOL. The results indicate that management of severe hypoglycemia and prevention of complications is crucial. Interventions should be developed to enhance coping abilities to improve efficacy for self-management for those with diabetes and promote their acceptance of the disease.

## 1. Introduction

Type 1 diabetes mellitus (DM) is an autoimmune disease that includes characteristics that lead to complete insulin deficiency due to immunological destruction of beta cells [1]. People with type 1 DM require insulin and must use it throughout their lives. The condition impacts their lifestyle and daily living in many ways. For example, special activities such as diet control and exercise are required to maintain a healthy lifestyle and prevent complications from the disease [2].

Reports indicate that the incidence of childhood-onset type 1 DM has been reported worldwide [3]. The incidence of type 1 DM among children is high in Europe and the USA and relatively low in Asia, including Korea. However, in Korea, the overall prevalence of childhood-onset type 1 DM has increased; per 100,000 persons, it was 32.85 in 2007 and 41.03 in 2017 [3]. Therefore, at a time when the prevalence of type 1 DM is increasing, it is necessary to examine the problems that it can have. Type 1 diabetes causes people to make changes to their lifestyle that can affect their entire family. It also affects their daily emotions and quality of life (QOL), including their food choices, blood sugar measurements, and injections [4]. Type 1 DM is primarily an autoimmune disease currently incurable. Hence, it is necessary to consider the QOL of diabetic people as they are required to live with and manage the disease for a lifetime [5].

QOL is a multidimensional concept defined by the World Health Organization as “an individual’s perceptions of their position in life, in the context of the culture and value systems in which they live, and in relation to their goals, expectations, standards, and concerns” [6]. QOL includes complex aspects of life, and there are several factors influencing the QOL of people with type 1 DM. First, objective disease-related characteristics, such as blood glucose level, glycated hemoglobin, complications, period of prevalence, and age of diagnosis affect their QOL. In particular, complications arise when blood vessels and nerves are affected by the progression of diabetes, and may occur in the form of cardiovascular complications, neurological complications, diabetic retinopathy, and diabetic foot, which can be fatal [7]. People with type 1 DM experience long-term stress, which is associated with multicomponent management and concerns of complications [8], and acute and chronic complications can be considered factors that influence QOL. Furthermore, besides disease-related factors such as level of disease management, various psychosocial factors resulting from managing the disease over an extended period can also influence QOL. It was also reported that psychosocial responses to type 1 DM, such as depression, anger, and stress, have the same effect on QOL as other factors, which include self-efficacy for self-management, coping, and family support [9]. Moreover, acceptance of the disease has been reported as factor that influences prognosis and people’ adaptation to the disease [10]; therefore, QOL is impacted by the way in which people recognize and accept lifestyle patterns or the disease and the difficulties associated with its management. Since the patient’s psychological condition is considered for diseases, the manner in which people accept the difficulties of managing a disease and the associated changes in lifestyle patterns affects their QOL [11,12]. Similarly, people with type 1 DM are known to have low QOL due to physical and psychological hardships arising from disease management [13].

However, there is a lack of research in South Korea on the factors associated with type 1 DM that affect QOL. Type 1 DM can be considered from the cultural perspective because it requires lifelong management. From a cultural point of view, unlike Western family culture, which emphasizes children’s independence and autonomy, in Korea, parent–child interdependence is important, and overprotection of children is a familiar family trait affecting QOL [14]. Therefore, it is necessary to investigate the quality of life of type 1 DM patients in South Korea. Since type 1 DM prevails for a longer period, and also appears at a fairly early age, it requires lifelong management. The management of type 1 DM is affected not only by insulin injections but also by the management of diet, exercise, activities of daily living, and stress [15]. Owing to such complex management strategies, efficacy for self-management can be expected to affect quality of life. The management of type 1 DM is affected not only by insulin injection but also by the management of dietary patterns, exercise, activities of daily living, and stress, in addition to relationships with healthcare providers, acquaintances, and family. As such, there are studies on type 1 DM’s relationships with QOL and the factors affecting QOL in living with it, but further consideration of new influencing factors is needed. For example, there is no research about factors influencing a patient’s acceptance of the disease. Although children and adolescents with type 1 DM must live with a demanding treatment regime, overall results of prior studies reveal that their generic QOL is not impaired compared to healthy peers [15]. The results of a previous study called indicate QOL is affected more by how one accepts the disease rather than by the disease itself. Therefore, it is necessary to help people in accepting and coping with the disease as much as possible. As type 1 DM develops at a young age, it is necessary to investigate the extent to which the disease can be completely tolerated, how well it can be managed, and the effect of the efficacy aspect on QOL. Therefore, the purpose of this research was to investigate the relationships between disease acceptance and self-management efficacy on QOL. The research questions of this study are as follows.

(1) What are the relationships between participant’s characteristics on their QOL?
(2) What are the relationships between disease acceptance, efficacy for self-management of diabetes and QOL?

This study, therefore, aims to investigate the factors influencing the QOL of people with type 1 DM.

## 2. Materials and Methods

This is a cross-sectional descriptive study designed to verify the factors influencing the QOL of people with type 1 DM.

### 2.1. Participants

The participants of this study were people with type 1 DM who belonged to a type 1 diabetes online community forum. The selection criteria were people who have type 1 diabetes, are 10 years of age or older, can manage their own diabetes, and can respond to questionnaires by themselves. Exclusion criteria were being hospitalized and treated for diabetes or other diseases, having cognitive or psychiatric disorders, and being unable to access and respond to the online questionnaires.

To calculate the number of participants required for this study, the free Statistics Calculators ver. 4.0 [16] was employed to ascertain the minimum sample size required to perform hierarchical linear regression [17] using the following parameters: nine independent variables in Block 1, two independent variables in Block 2, a two-sided significance level (α) of 0.05, statistical power (1-β) of 0.9, and medium effect size (f^2^) of 0.15 [18]. The required number of participants, accounting for a 10% withdrawal rate, was computed as 107. An online survey with a consent form was shared with the type 1 DM online community forum, which is available only to approved and registered members. Efforts were made to avoid potential bias in participant recruitment. Ultimately, a total of 111 participants participated, and no participants withdrew.

### 2.2. Assessments

#### 2.2.1. Characteristics of the Participants

To identify the sociodemographic and disease-related characteristics of the participants, their age, sex, religion, duration of disease, experiences of hypoglycemia in the past one month, frequency of hypoglycemia average per month, blood glucose level at the time of hypoglycemia for past one month, presence of complications, and recently measured glycated hemoglobin (HbA1C) values were collected. The categorical variable, sex, was answered as “male” or “female”; religion, presence of complications, and experience of hypoglycemia for the past one month were answered as “yes” or “no”.

#### 2.2.2. Quality of Life

The QOL of type 1 DM people was measured using the Korean version of the PedsQL^TM^ 3.0 Diabetes Module developed by Varni et al. [19] and tested by Han et al. [20] for its reliability and validity. The PedsQL^TM^ 3.0 diabetes module is a validated and developed tool for children, adolescents, and early adults [21]. In this study, a tool that is applicable for a wide range of participants was chosen, as we needed one tool that could examine QOL for participants of varying age groups. When using the same item for adults, it was used after being reviewed by an expert group (two nursing professors, one endocrinologist, two diabetes specialists) to see if there is any problem with content validity. The PedsQL^TM^ 3.0 Diabetes Module consists of 5 subdomains (diabetes symptoms, treatment barriers, treatment adherence, worry, and communication) and 28 items. Each QOL item was scored on a 5-point scale ranging from 0 (never) to 4 (almost always); items were reverse scored and linearly transformed to a 0–100 scale as follows: 0 = 100, 1 = 75, 2 = 50, 3 = 25, 4 = 0. The total mean score ranges from 0 to 100, with a higher score indicating better QOL. The reliability (Cronbach’s α) of the PedsQL^TM^ 3.0 Diabetes Module was 0.89 when it was developed by Han et al. in Korean and 0.92 in the present study.

#### 2.2.3. Self-Efficacy for Diabetes Self-Management

The self-efficacy scale for diabetes self-management was developed by Iannotti et al. [22], were designed and tested their validity for young children by Allen et al. [23], and tested by Boo et al. [24] for its reliability and validity in Korean. This tool consists of ten items (six items on blood glucose testing, insulin injections, and life, and two items each on diet and exercise). Each item was measured on a scale from 0 (no confidence at all) to 10 (as confident as possible). The mean score ranged between 0–10, and the higher the score, the higher the self-efficacy for diabetes self-management. The reliability (Cronbach’s α) of this scale was 0.86 when it was developed by Boo et al. in Korean and 0.89 in the present study.

#### 2.2.4. Acceptance and Action Questionnaire (AAQ)

The AAQ was developed by Bond and Bunce [25], tested by Moon [26] for its reliability and validity in Korean, and were used and tested for young children by Choi and Kim [27]. This tool consists of 16 items, each rated on a 7-point scale from 1 (never true) to 7 (always true). High scores on the AAQ are reflective of greater experiential avoidance and immobility, while low scores reflect greater acceptance and action. The reliability (Cronbach’s α) of this scale was 0.82 when it was developed by Moon in Korean and 0.84 in the present study.

### 2.3. Ethical Considerations

The researchers obtained approval for the research plan from the institutional review board of E University (no. EU16-04). All participants signed an informed consent form, which included a description of the study. Finally, all participants were informed of their rights under the Declaration of Helsinki.

The participants were informed of the purpose and procedure of the study, as well as of their rights, and were guaranteed confidentiality. Additionally, only those individuals who voluntarily consented to participate in the study after reading the online consent form were allowed to take the survey. Participants who were children (under the age of 18) with type 1 DM were asked to participate in the survey only if a guardian’s or parent’s consent was provided.

### 2.4. Data Collection

The data were collected from 8 October 2016, to 24 May 2017, through an online self-report questionnaire. The researchers explained the purpose and procedures of the research to the administrators of a type 1 DM online community forum for people with type 1 DM, requesting their cooperation and consent. An online survey for recruiting participants was posted on the website of the type 1 DM online community forum. Characteristics of the participants, QOL, self-efficacy for diabetes self-management, and responses to an acceptance and action questionnaire were collected.

### 2.5. Statistical Analysis

The collected data were analyzed using the PASW Statistics program (version 23.0, AMOS 20.0, SPSS, IBM, USA). The characteristics of the participants and the mean (SD) values of QOL were analyzed. The normality of the variables was measured by Kolmogorov–Smirnov test, and outcome variables showed a normal distribution. In addition, differences in QOL according to the characteristics were analyzed using an independent *t*-test. Pearson’s correlation was used to examine the correlation among the efficacy for self-management of diabetes, AAQ, and QOL of people with type 1 DM. Factors influencing the QOL of people with type 1 DM were identified through hierarchical multiple regression. The hierarchical multiple regression models were estimated, first with all the variables in Block 1 (demographics and disease characteristics), followed by self-efficacy for diabetes self-management and acceptance and action as predictors in Block 2. The cutoff for statistical significance in the present study was *p* < 0.05.

## 3. Results

### 3.1. Characteristics of the Participants

The participants in the present study comprised 111 people with type 1 DM aged 28.42 (*SEM* 1.11) years, including 71 (64.0%) females, and 68 (61.3%) non-religious participants. Nearly all of the participants (*n* = 105, 94.6%) experienced hypoglycemia on an average of 5.58 (*SEM* 0.56) times per month, and the mean blood glucose level for all participants combined was 56.38 (*SEM* 1.06) mg/dL, and their serum HbA1C was 7.9% (1.9%). The mean duration of disease was 10.27 (*SEM* 0.71) years, and 22 (19.8%) participants had experienced complications. The average scores of self-efficacy for diabetes self-management (range: 0–10) and experiential acceptance and effective action orientation (range: 1–7) were 5.47 (*SEM* 0.18) and 3.94 (*SEM* 0.07), respectively (Table 1).

### 3.2. Descriptive Statistics of QOL

The average score of QOL was 59.16 (*SEM* 1.56). Regarding the five subcategories, the average score of diabetes symptoms was 60.24 (*SEM* 1.49); treatment barriers, 59.80 (*SEM* 2.02); treatment adherences, 59.20 (*SEM* 2.06); worry, 50.00 (*SEM* 2.39); and communication, 63.44 (*SEM* 2.74) (Table 2).

### 3.3. Differences in the QOL by Characteristics of the Participants

With respect to QOL, a duration of disease less than 10.27 years (*t* = −2.24, *p* = 0.027), no complications (*t* = −2.44, *p* = 0.016), serum HbA1C less than 7.92 % (*t* = 2.61, *p* = 0.010), self-efficacy for diabetes self-management score more than 5.47 (*t* = −7.33, *p* < 0.001), and acceptance of disease and effective action score more than 3.94 (*t* = −6.23, *p* < 0.001) were found to be statistically significant (Table 3).

### 3.4. Correlation of QOL and Other Variables

QOL was positively correlated with self-efficacy for diabetes self-management (*r* = 0.73, *p* < 0.001) and acceptance and action (*r* = 0.54, *p* < 0.001). Furthermore, self-efficacy for diabetes self-management and acceptance and action were positively correlated (*r* = 0.47, *p* < 0.001; Table 4).

### 3.5. Factors Influencing QOL

To identify factors influencing the QOL of people with type 1 DM, a hierarchical multiple regression analysis was conducted with blood glucose level at hypoglycemia, and serum HbA1C that can affect quality of life as continuous variables; the categorical variable of complications showing differences in quality of life in univariate analysis was used as dummy variables in the first step. The duration of disease showed a significant difference in quality of life in univariate analysis but was highly correlated with complications. Of the two variables, only the complication variable entered the regression model, excluding the duration of disease. Subsequently, self-efficacy for diabetes self-management and acceptance and action were included, revealing a significant correlation found in the analysis in the second step (Table 5).

When the participants’ characteristics were used as the input variables of Model 1 in the input step, identifying factors influencing QOL, there was no multicollinearity problem among the independent variables. The factors found to influence the QOL of people with type 1 DM in Model 1 were blood glucose level at hypoglycemia (B = 0.41, *t* = 2.21, *p* = 0.030) and complications (B = −8.22, *t* = −2.01, *p* = 0.048), with an explanatory power of 9.5% for the QOL variance (*F* = 3.78, *p* = 0.014). In Model 2, we used the participants’ characteristic variables, and included self-efficacy for diabetes self-management (B = 5.06, *t* = 2.37, *p* = 0.020) and acceptance and action (B = 5.72, *t* = 7.66, *p* < 0.001) in the final input step; these variables were found to have a significant effect on QOL. Overall, four variables were found to exert a significant effect on QOL (blood glucose level at hypoglycemia and complications in Model 1 and self-efficacy for diabetes self-management and acceptance and action in Model 2), and all the variables explained 57.3% of the variance in QOL (*F* = 22.24, *p* < 0.001).

## 4. Discussion

When the factors influencing the QOL of people with type 1 DM were analyzed, blood glucose level at hypoglycemia and the presence of complications exhibited statistical significance among the general and disease-related characteristics of the participants. Furthermore, these explained their QOL by 9.6%. Participants’ QOL was lower in proportion to the blood glucose level at hypoglycemia, which indicates that having severe hypoglycemia lowers the QOL of people. In other words, experiencing hypoglycemia can be fatal for patients, which supports the findings of a previous study [28], which revealed that the fear of having hypoglycemia lowers QOL. This result supports the suggestion that fear of hypoglycemia is a common problem for individuals with diabetes and can produce a negative impact on QOL in individuals with type 1 DM [29]. Severe hypoglycemia occurs when the blood glucose level is 40–50 mg/dL or below [30]. When a patient experiences hypoglycemia, their level of stress hormones, such as cortisol and adrenaline increase, and that leads to physical arousal, shivering, chills, blurred vision, and dizziness [31] and adversely affects patients, thus reducing their QOL. While the frequency of experiencing hypoglycemia was not statistically significant, the low blood glucose level at hypoglycemia did affect QOL. Therefore, the severity of hypoglycemia has a greater effect on QOL than the frequency of experiencing hypoglycemia, which implies that interventions are needed to prevent the occurrence of severe hypoglycemia.

Furthermore, participants with complications had lower QOL in this study, indicating that the occurrence of complications has a negative influence on QOL. Some of the complications of diabetes include neuropathic pain, mental disorders, cardiac diseases, and functional impairments such as foot ulcers or sexual dysfunction [32]; physical pain or discomfort associated with these complications reduces QOL. People with type 1 DM have the potential risk of developing severe microvascular or macrovascular complications. Thus, complications should be prevented or detected early by testing blood glucose, controlling insulin levels, undertaking periodic visits to the hospital, eye examinations, monitoring blood pressure, and conducting microalbuminuria tests [7]; proper management is required in the case of complications.

The results examining the psychosocial aspect imply that there is psychological acceptance of the disease, but there was a difference according to the existence of religion [33,34] and the degree of awareness of the importance of religion [35]. We also considered differences according to the presence or absence of religion. Whether they acknowledge any religion and how important it is to them seems more relevant than the type of religion a participant may practice; there were no significant differences associated with religion. Because this study investigated the factors that ultimately affect the QOL, the presence or absence of religion does not seem to directly affect the QOL.

As there may be sex-related differences in the psychosocial aspects of disease progression or acceptance, we analyzed whether there are differences in QOL according to sex. The results indicate no difference in overall QOL according to sex; this finding is consistent with those of a study by Lee et al. [36]. However, there may be some characteristics related to sex that have not been examined that impact QOL for people with type 1 DM. Therefore, future research should include an in-depth examination of sex-specific aspects, such as the onset of menstrual cycle and other developmental-stage related aspects. Overall, a total of four variables, including self-efficacy for diabetes self-management, acceptance and action, blood glucose level at hypoglycemia, and the presence of complications, explained QOL by 57.3%. Since the explanatory power increased when self-efficacy for diabetes self-management and acceptance and action were included, it is clear that they have a significant effect on QOL. The former refers to the confidence of people with type 1 DM that they can manage the disease in the diverse situations that they may experience. Hence, having such capabilities leads to higher QOL. A higher efficacy for self-managing a disease also represents the capacity to handle hypoglycemia or emergences, and cope with diet restriction, exercise, medication, and other treatments, wherein such confidence and belief contribute to improving QOL. The finding that higher efficacy for the self-management of diabetes leads to higher QOL corresponds to the results of previous studies, which reported that people with diabetes feel anger and shame for inadequate self-management; these feelings lead to loss of motivation to practice self-management, which ultimately affects their QOL [37], as well as of those which reported that self-efficacy influences QOL [7]. In a previous study, children with type 1 DM were seen to especially face challenges related to diabetes self-management at school, which included lack of awareness of diabetes among teachers and students, paucity of time to conduct blood glucose test and inject insulin, lack of space for insulin injection that guarantees privacy and safety, and the ability to cope with hypoglycemia during classes [38]. Thus, the low QOL of people with type 1 DM is not simply associated with having to inject insulin to control blood glucose levels but also with the difficulties faced in everyday life. A previous study [31] reported that lifestyle interventions, including diet or physical activity, can improve diabetes outcomes and QOL. Thus, interventions are needed to help those with diabetes improve their ability to cope with stress and manage their blood glucose level in various situations.

The finding that better acceptance of the disease leads to higher QOL corresponds to the findings of a study that reported that such receptiveness is related to QOL [39]. Readily accepting a disease signifies a proper understanding of the disease and the ability to recognize one’s conditions with sensitivity, which promotes management of the disease without repulsion. Thus, complete acceptance of a disease that requires lifelong management and having a positive attitude toward disease management were found to have a positive effect on QOL. A previously developed health model for children or adolescents with type 1 DM suggested some processes of cognitive appraisal of stressful situations and willingness to cope with such situations, which showed that the manner in which stress is handled is more important than the stressful situations themselves [7]. Additionally, it is important for people with type 1 DM to face and accept the social perception of the disease to ensure sound management of the disease [5]. As positive psychological factors, such as subjective well-being, are closely related to the QOL of children and adolescents with chronic diseases [40], people need to be supported to accept the disease.

Thus, preventing acute complications such as hypoglycemia and chronic complications that may develop over time, improving self-efficacy for disease management, and developing a deeper understanding of the disease will help people come to terms with their diagnosis and improve the QOL of people living with type 1 DM. This study is particularly significant to nursing practice because it further enriches our conception of people living with type 1 DM. The results of this study demonstrate that full acceptance of their diagnosis ultimately improves participants’ QOL, and through the assessment of what aspects are difficult to accept, we will be better able to assist participants in coming to terms with living with type 1 DM. To achieve this goal, it is necessary to manage the care of type I DM in a holistic manner.

There were limitations in this research that must be acknowledged, but they can be addressed by future studies. First, the relationships found in this study do not imply causal relationships. Second, it is necessary to consider more variables or potential confounding variables. Third, it is necessary to develop an instrument that can consider various aspects of the stage of growth and development of a participant’s type 1 DM. Third, research is needed to measure disease acceptance in a way that further clarifies the concept. Last, longitudinal research is needed to provide tailored care for children of all ages with type 1 DM.

## 5. Conclusions

Factors affecting QOL need to be examined as they are particularly important for those with diseases requiring lifelong management, such as type 1 DM. The results of examining the factors affecting the QOL people with type 1 DM showed that the severity of hypoglycemia, presence of complications, efficacy for self-management of diabetes, and acceptance of the disease explained QOL by 59.0%. Therefore, interventions should be provided to prevent people living with the condition from descending into severe hypoglycemia, and further complications due to long-term hyperglycemia should be prevented. To improve the efficacy of self-management for people with diabetes, their ability to cope with various situations in everyday life need to be enhanced. Thus, from the standpoint of providing comprehensive nursing care to patients, it is necessary to help people fully accept the disease to improve their QOL.

To achieve this goal, it is necessary to develop interventions and practices that help patients better understand common difficulties people face in coming to terms with the disease, so that they may come to fully accept and live with the disease. One of the limitations of this study is that the analyzed aspects of QOL were limited to aspects of disease management and attitudes toward diseases. Therefore, in addition to the factors examined in this study, other factors such as medication, smoking, age variation, sex, socioeconomic status, maturation level, social/family support network, access to healthcare, information/education on type 1 DM management that affect QOL should be investigated in future studies. Another limitation is that we did not include psychological status, which is a factor that affects quality of life.

Hypoglycemia and the presence of complications were factors affecting the QOL of people with type 1 DM. In the case of hypoglycemia, which is a short-term complication, our results indicate that treatment priority should be given to preventing the severity of hypoglycemia than the frequency of hypoglycemia. Education and training, preventing and remediate severe hypoglycemia, should be considered crucial when designing the nursing education curriculum.

## Figures and Tables

**Table 1:** Characteristics of the Participants (*N* = 111).

Characteristics	*n* or M	% or *SEM*
Age (year)	28.42	1.11
Adolescent (<19)	28	25.2
Adult (≥19)	83	74.8
Sex		
Male	40	36.0
Female	71	64.0
Religion		
No	68	61.3
Yes	43	38.7
Duration of disease (year)	10.27	0.71
<10	66	59.5
≥10	44	39.6
Experience of hypoglycemia		
No	6	5.4
Yes	105	94.6
Frequency of hypoglycemia (average per month)	5.58	0.56
<5.58	78	70.3
≥5.58	33	29.7
Blood glucose level at hypoglycemia (mg/dL, past one month) *	56.38	1.06
<56.38	41	36.9
≥56.38	44	39.6
Complications		
No	89	80.2
Yes	22	19.8
HbA1C (%) *	7.92	0.18
<6.5	18	16.2
≥6.5	87	78.4
Efficacy for self-management of diabetes	5.47	0.18
<5.47	52	46.8
≥5.47	59	53.2
Acceptance and action	3.94	0.07
<3.94	53	47.7
≥3.94	58	52.3

Note. * Missing value. M = Mean; *SEM*: Standard error mean; *n* = Number of participants; HbA1C = Glycated hemoglobin.

**Table 2:** Descriptive Statistics of QOL (*N* = 111).

Variable	M *(SEM)*	Min–Max (0–100)
QOL	59.16 (1.56)	13.39–98.21
Diabetes symptoms	60.24 (1.49)	18.18–97.73
Treatment barriers	59.80 (2.02)	6.25–100.00
Treatment adherences	59.20 (2.06)	7.14–100.00
Worry	50.00 (2.39)	0.00–100.00
Communication	63.44 (2.74)	0.00–100.00

Note. M = Mean; *SEM*: Standard error mean; QOL = quality of life; Min–Max = Minimum–Maximum.

**Table 3:** Difference of QOL according to Characteristics of the Participants (*N* = 111).

Characteristics	QOL	
	M (*SEM*)	*t* (*p*)
Age (year)		
Adolescent (<19)	60.75 (3.51)	0.59 (0.558)
Adult (≥19)	58.63 (1.73)	
Sex		
Male	61.21 (2.81)	0.98 (0.328)
Female	58.01 (1.86)	
Religion		
No	59.77 (1.85)	0.49 (0.627)
Yes	58.20 (2.79)	
Duration of disease (year)		
<10	62.01 (1.92)	2.23 (0.028)
≥10	54.97 (2.59)	
Experience of hypoglycemia (past one month)		
No	54.36 (6.67)	−0.58 (0.563)
Yes	59.38 (1.61)	
Frequency of hypoglycemia (average per month)		
<5.58	60.98 (1.89)	1.81 (0.074)
≥5.58	54.87 (2.67)	
Blood glucose level at hypoglycemia (mg/dL, past one month) *		
<56.38	57.88 (2.02)	1.32 (0.190)
≥56.38	62.68 (3.01)	
Complications		
No	61.02 (1.70)	2.44 (0.016)
Yes	51.66 (3.48)	
HbA1C (%) *		
<6.5	57.64 (2.62)	−0.49 (0.627)
≥6.5	59.22 (1.86)	
Efficacy for self-management of diabetes		
<5.47	49.14 (2.06)	−7.33 (<0.001)
≥5.47	67.99 (1.59)	
Acceptance and action		
<3.94	50.39 (2.08)	−6.23 (<0.001)
≥3.94	67.18 (1.68)	

Note. * Missing value. QOL = Quality of life; M = Mean; *SEM*: Standard error mean; HbA1C = Glycated hemoglobin.

**Table 4:** Correlation of the Variables (*N* = 111).

Variable	QOL	Efficacy for Self-Management of Diabetes	Acceptance and Action
	r (*p*)		
QOL	1		
Efficacy for self-management of diabetes	0.73 (<0.001)	1	
Acceptance and action	0.54 (<0.001)	0.47 (<0.001)	1

Note: QOL = Quality of life; r = correlation coefficient.

**Table 5:** Influencing Factors on QOL (*N* = 111).

Variable	Model 1			Model 2		
	B	SE	*t* (*p*)	B	SE	*t* (*p*)
Intercept	48.81	12.84	3.80 (<0.001)	5.39	10.79	0.50 (0.619)
Blood glucose level at hypoglycemia (mg/dL)	0.41	0.18	2.21 (0.030)	0.04	0.14	0.27 (0.786)
Complications (ref. = No)	−8.22	4.09	−2.01 (0.048)	−4.46	2.84	−1.57 (0.121)
HbA1C (%)	−1.22	0.99	−1.24 (0.221)	0.17	0.69	0.24 (0.808)
Efficacy for self-management of diabetes				5.06	2.13	2.37 (0.020)
Acceptance and action				5.72	0.75	7.66 (<0.001)
*F* (*p*)	3.78 (0.014)			22.24 (<0.001)		
Adj.R^2^	0.095			0.573		
Tolerance	0.978~0.994			0.731–0.958		
VIF	1.006~1.022			1.043–1.368		
Durbin-Watson	2.036			2.469		

Note. QOL = Quality of life; Adj.R^2^ = Adjusted R^2^; VIF = Variance Inflation Factors.

## Data Availability

Data sharing not applicable.
